# Scraps

**Published:** 1897-06

**Authors:** 


### SCRAPS

PICKED UP BY THE ASSISTANT-EDITOR.

**Clinical Records** (19).—The *Guyoscope* is responsible for the following:—

There was a young lady called Margery,
Whose head was a walking menagerie.
Folks said, "You should wash,"
But she answered, "Oh, bosh,
I'll apply some Unguentum Hydrargyri."

**Medical Philology** (XXII.).—The *Promptorium* has "COGHE. *Tussis.*" The Heber MS. has "cowhe, or host," which was followed by Wynkyn de Worde in his 1516 edition. Pynson in 1499 printed "cough or horst." As the English equivalents of *tussio* and *tussito*, other readings give cowyn or hostyn, cowhyn, and cowghen. The *Promptorium* also has "HOOSE, or cowghe. *Tussis*," with cowgth as a variant spelling. In addition we find "HOSTYN', or rowhyn', or cowghyn. *Tussio, tussito*," with further varieties of spelling as rowwhyn and rewyn.

The *Catholicon* has "an **Host**; *tussis, tussicula*," and "to **Host**; *tussire*."

The notes by Mr. Way and Mr. Herrtage include the following:—

Among the virtues of "horhowne," as stated in a translation of Macer's Treatise on Plants, MS. XVth Cent. belonging to Hugh W. Diamond, Esq. is the following: "þis erbe y-dronke in olde wyne helpiþ þe kynges hoste, and þe comone coghe eke." In another place a decoction of roots of "skyrewhite" is recommended to heal "þe chynke and þe olde coghe." Skinner says the hooping-cough was termed in Lincolnshire kin-cough, and derives the word from the Belg. kinckhost, and the verb kinchen, *difficulter spirare*.

"*Tussis*, host." Vocab. Roy. MS. 17 C. XVII. "*Raucedo*, hoocenesse; *raucidus*, hooce; *raucidulus*, sum dele hoce; *raucus*, hoost," MED. Forby gives hoist, a cough. Ang. Sax. hwosta, *tussis*.

"*Yvresce fait fort home chatouner* (creopen),
*Home aroee* (hoos) *fait haut huper* (ȝellen)." G. de Bibelesw.

The Craven dialect still retains the word hoste, hoarseness.
Chaucer, Miller's Tale, 3697, tells us how Absolom when he went to serenade Alison—

"Softe he *cowhith* with a semysoun"

"*Tussis.* The cowhe." Medulla.
"His ene wes how, his voce wes hers *hostand.*" Henrysone, Bannatyne Poems [1571] p. 131, in Jamieson, who also quotes from Dunbar, Maitland Poems, p. 75.

"And with that wourd he gave ane *hoist* anone."

English words ending in *ough* are well known to be a terrible difficulty to the foreigner, who may see from these notes that "cough," was at one time pronounced "cow," and that it had to go through many changes before its present spelling was fixed.

"Host" as meaning cough is clearly from the Anglo-Saxon *hwosta.* Cough in modern German is *husten.* Jamieson, in addition to the instance quoted above, gives "host" and "hoast"; "hoase" and "hoast" appear in the English Dialect Society's *Rutland Words*, 1891.

"Hoarse" is allied to the foregoing; being connected with the Anglo-Saxon *hás* and appears in Middle-English in various forms. In *Piers Plowman* (ed. Wright, p. 367, l. 12017) is "hoors in the throte," and Chaucer (ed. Morris, v. 165, *Boke of the Duchesse*, 347) has "horse of soune."

Mr. Way's note brings out some interesting points in connection with "chincough," a term still often used for whooping cough.

As equivalents of whooping cough, "kink-hoast," "kin-couf," and "kin-cough" all appear in *Northumberland Words*, issued by the Dialect Society in 1894. Chincough is clearly allied to these and to various Continental forms, which are given by the *New English Dictionary* as containing the Saxon stem, *kink*, to gasp or pant. Chincough, which it is obvious should be pronounced kincough, is therefore a cough specially associated with panting.

The *chin* of "chincough" was sometimes connected with *chine*, a word which, meaning a cleft or fissure, had lived on in various forms of spelling from Saxon times until the 16th century, when "it was superseded, except in the local use" of the Isle of Wight and Hampshire coast, by *chink.* If this connection of *chin* with *chine* had no etymological justification it had, at all events, some pathological basis, for it might have been supposed to denote the condition of things during the paroxysm of whooping cough when the air was passing through a narrowed opening—a chine or chink.

Neither *chink* nor *chincough* has been met with earlier than the 16th century.

Popular etymology, which sometimes associated the word with "chin" is, according to the *New English Dictionary*, also responsible for the form "kynges hoste," which appears in one of Mr. Way's quotations. This falsely connected "chin" with "king." Under the heading of Nomina Infirmitatum in a 15th Century Pictorial Vocabulary printed by Wright is "*Hec reuma, An*^ce^ a chynge." *Ch* and *k* would be easily interchangeable. King in Anglo-Saxon was cyning, or cyng, or cyneg.

**Medical "Bits of Old Bristol"** (3).—The order given by the Bishop of Worcester at his Visitation of the Abbey of St. Augustin in 1278 (see March number of *Journal*, p. 103) does not seem to have produced all the results that were desired. At another Episcopal Visitation in 1320 a further order was made "that the sick be better provided for." (Barrett, p. 263.)

This probably referred more to general care than to medical treatment. It would seem that the occupants of the infirmary did not always get the good things which were intended for them, but that their better food was sometimes appropriated by the monks who were in good health. Fosbrooke (*British Monachism*, ed. 1817, p. 324) quotes a passage from *Piers Plowman* as an allusion to the special dishes prepared for the patients in the infirmary. Do-wel (B-Text, E.E.T. Soc. ed., xiii. 105-9) says:

"By þis day, sire doctour," q*uod* I · "þanne be ȝe nouȝt in dowel;

For ȝe han harmed vs two · in þat ȝe eten þe puddyng,

Mortrewes, and other mete · and we no [morsel] hade!

And if ȝe fare so in ȝowre fermorie · ferly me þinketh,

But chest be þere charite shulde be · & ȝonge childern dorste pleyne!"

The parallel passage in the C-Text (E.E.T. Soc. ed., xvi. 115-9) is:

> "Certes, sire," þanne seide ich · "hit semeþ nat here,

> In þat ȝe parteþ nat with ous poure · þat ȝe passeþ dowel,

> Noþer louyeþ as ȝe lereþ · as oure lorde wolde,

> And ȝe fare þus with ȝoure sike freres · ferly me þynkeþ

> Bote dowel endite ȝow · *in die iudicii.*"

Fosbrooke gives only the first of the passages, merely with the intention of showing that it was the custom to provide better food for the inmates of the infirmary. But it is also clear that the speaker meant to make a charge that this was consumed by those for whom it was not intended. Fosbrooke evidently missed the meaning of the word "fare," which at first sight might be thought to have a definite reference to food, but that is a later use, and in earlier times it meant "do" or "go," the meaning which it now holds in "farewell," "thoroughfare," and "welfare."

This was probably only one of the ways in which the "sike freres" were unfairly treated. Bearing on this subject, Skeat (E.E.T. Soc. ed., Part IV., i. 307) alludes to l. 614 of P. Pl. Crede and his note on it. I regret I am not able to quote this, as I have not seen that edition of the Crede.

It may be some consolation to the profession of to-day to remember that the person addressed in the first line of the first passage quoted was, of course, not a medical doctor.

Of the therapeutic measures adopted bleeding was the most frequent. In some abbeys there was a bleeding house called Flebotomaria. The operation, which was performed by a servant, as mentioned in our March number, was termed "minution." In the *Journal* for March and June, 1893, pp. 72, 144, I quoted various notes about the practice of bleeding in the Middle Ages. There were certain festivals when bleeding was not allowed in the monasteries, for instance at All Saints because on the morrow all the priests were to celebrate masses for the dead. In the order of S. Victor, which was also under the Augustinian rule, the brethren were bled five times a year—in September, before Advent, before Lent, after Easter, and after Whitsuntide. Anyone who wishes to learn more about these monastic bleedings will find copious references in Du Cange s.v. *minuere.*

It was thought necessary that after a bleeding the patient should be put on extra diet. The writer of the *Ancren Riwle*—a work written in the thirteenth century for the guidance of the nuns of a house in Dorsetshire—says, "Two maner men habbeð neode uorte eten wel, & forto drinken wel—swinkinde men, & blod-letene"; a passage which is thus rendered by Morton in the Camden Society edition of the work, pp. 260-1, "Two sorts of men have need to eat and to drink well—men who labour, and men who have been let blood." Monks who had been bled were admitted to the infirmary for a short time after the operation, and in order to get a chance of the better living supplied for its occupants they frequently manifested a desire for venesection; and this no doubt was one of the purposes for which they feigned sickness, and one of the abuses to which attention was called in the order made by the Bishop in 1278 (see March *Journal*, p. 103).

In prescriptions found in works about the period in question many nauseous ingredients were often introduced. The sick monks doomed to suffer many an injustice had probably a fortunate escape from practical experience of pharmacy of this kind. In the monastic infirmary there was not likely to be much administration of drugs. Fosbrooke quotes Burton who, writing in 1621, said "that there was of old no use of physick amongst us, and but little at this day, except it be for a few nice idle citizens, surfeiting courtiers, and stall-fed gentlemen lubbers. The countrey people use kitchen physick; and common experience tells us, that they live freest from all manner of infirmities, that make least use of apothecaries physick. Many are overthrown by preposterous use of it, and thereby get their bane, that might otherwise have escaped." (*Anatomy of Melancholy*, ed. 1806, ii. 86.)

